# A Single-Cell Atlas of the Atherosclerotic Plaque in the Femoral Artery and the Heterogeneity in Macrophage Subtypes between Carotid and Femoral Atherosclerosis

**DOI:** 10.3390/jcdd9120465

**Published:** 2022-12-16

**Authors:** Ping Wang, Lin Zheng, Maolin Qiao, Tianliang Zhao, Ruijing Zhang, Honglin Dong

**Affiliations:** 1 Department of Vascular Surgery, The Second Hospital of Shanxi Medical University, Taiyuan 030001, China; 2 Department of General Surgery, The Second Hospital of Hebei Medical University, Shijiazhuang 050001, China; 3 Department of Nephrology, The Second Hospital of Shanxi Medical University, Taiyuan 030001, China

**Keywords:** atherosclerosis, peripheral arterial disease, plaque heterogeneity

## Abstract

Atherosclerosis of femoral arteries can cause the insufficient blood supply to the lower limbs and lead to gangrenous ulcers and other symptoms. Atherosclerosis and inflammatory factors are significantly different from other plaques. Therefore, it is crucial to observe the cellular composition of the femoral atherosclerotic plaque and identify plaque heterogeneity in other arteries. To this end, we performed single-cell sequencing of a human femoral artery plaque. We identified 14 cell types, including endothelial cells, smooth muscle cells, monocytes, three macrophages with four different subtypes of foam cells, three T cells, natural killer cells, and B cells. We then downloaded single-cell sequencing data of carotid atherosclerosis from GEO, which were compared with the one femoral sample. We identified similar cell types, but the femoral artery had significantly more nonspecific immune cells and fewer specific immune cells than the carotid artery. We further compared the differences in the proportion of inflammatory macrophages, and resident macrophages, and the proportion of inflammatory macrophages was greater within the carotid artery. Through comparing one femoral sequencing sample with carotid samples from public datasets, our study reveals the single-cell map of the femoral artery and the heterogeneity of carotid and femoral arteries at the cellular level, laying the foundation for mechanistic and pharmacological studies of the femoral artery.

## 1. Introduction

Cardiovascular disease (CVD) is the leading cause of death and disability in China and the world, seriously threatening human health. Among them, atherosclerosis, as a chronic inflammatory disease, causes the artery wall to thicken, stiffen and lose elasticity, which leads to ischemia and an inadequate blood supply to vital organs. Femoral atherosclerosis affects more than 200 million individuals worldwide and typically manifests as intermittent claudication but often with tissue loss and severe limb ischemia characterized by ulcers or gangrene at its most advanced stage. At the same time, patients with lower extremity atherosclerosis have a significantly increased risk of functional cardiovascular abnormalities [1]. There is evidence that within five years after diagnosing lower extremity atherosclerosis, 10 to 15 percent of people with intermittent claudication die of cardiovascular causes [2]. Therefore, early diagnosis and treatment to avoid the late symptoms and complications of femoral atherosclerosis are highly important. However, the early stage of femoral atherosclerosis is asymptomatic. The main clinical measures are endovascular treatment after the onset of symptoms or femoral artery endarterectomy if the stenosis is severe. Many patients with severe limb ischemia receive amputations due to lacking early diagnosis and detection. Therefore, it is essential to study the pathological mechanism of femoral atherosclerosis and screen patients with it as early as possible.

Atherosclerosis is a complex chronic disease with multiple stages. The main pathological mechanisms are endothelial cell dysfunction, macrophage polarization, inflammation, and immune responses [3,4]. Inflammatory mediators are critically involved in locally deposited lipids and the formation of foam cells, contributing to the formation of an atherosclerotic necrotic core and altering plaque thickness and stability [5]. In addition, studies have proven significant differences in the types and proportions of immune cell infiltration in atherosclerotic plaques from different peripheral artery sites [6]. The results of HE staining show that atherosclerotic plaques with more inflammatory macrophages and T-cell infiltration have poorer stability [7,8,9]. Therefore, identifying the pathological characteristics of atherosclerosis of different arteries has significant implications for the prevention and accurate targeted treatment of atherosclerosis and complications.

Although previous studies have analyzed cell types of carotid and coronary atherosclerosis [8,10,11,12], the analyses of arteries elsewhere are unsuitable for systemic atherosclerosis. Moreover, no study has studied the cellular composition of “atherosclerotic plaques” in the femoral artery. Therefore, this study started with single-cell sequencing of a femoral plaque, described the cellular composition and the immune cell subtypes of the femoral atherosclerotic plaque in detail and evaluated cell types associated with carotid and femoral plaques. Comparison can improve understanding of their heterogeneity of pathophysiology and open up new therapeutic targets.

## 2. Materials and Methods

### 2.1. Sample Collection

We collected four consecutive patients with severe common femoral artery stenosis and three with symptomatic carotid atherosclerotic plaques who were about to undergo endarterectomy. The patients underwent perioperative anticoagulation and antiplatelet therapy before surgery according to the guidelines for the diagnosis and treatment of lower extremity artery occlusion and guidelines for the diagnosis and treatment of carotid stenosis [13,14]. The basic patient information is provided in Supplementary file S1. The sample collection plan and exclusion standard were submitted to the Ethics Committee of the Second Hospital of Shanxi Medical University and approved, No. (2022) YX No. (006).

### 2.2. Plaque Processing and Quality Testing

The treatment of femoral plaques is performed rapidly after dissection from the femoral artery during the surgery. Samples from the narrowest part of the common femoral artery containing tissue with a calcified core and a small area of thrombus organization were taken, divided, and mechanically dissociated with eye scissors after being cleaned with cool PBS. Dissociated samples were kept in the tissue storage solution (Miltenyi, Bergisch Gladbach, Germany) and then immediately sent to the Lc. research facility for single-cell sequencing. After the tissue was treated with collagenase, a single-cell suspension was prepared, and strict quality control was performed.

A total of three samples were put on the machine, among which only one sample had a high cell number and cell viability. The remaining two samples had relatively few cells due to a large number of atherosclerotic necrotic tissues and the small number of active tissues, which meant they did not meet the quality standards. Detailed control information is provided in Supplementary file S2. A sample of femoral artery plaque with the desired cell count and viability yielded 14,351 cells.

### 2.3. Construction and Sequencing of Single-Cell Libraries

The suspension was used to generate single-cell gel beads in the emulsion. After reverse transcription, gel beads in an emulsion were disrupted. A total of 14,113 cells were tagged in one femoral plaque. Single Cell 3′ v2 libraries were generated following the Single Cell 3′ v2 Reagent Kits User Guide (Document CG00052, 10X genomics). The libraries were sequenced on the Illumina HiSeq Xten platform.

### 2.4. Bioinformatics Analysis Process of the Single-Cell Libraries

#### 2.4.1. Sequencing Data Quality Control and Quantification Based on CellRanger

Using 10X Genomics’ official analysis software CellRanger (https://support.10xgenomics.com/single-cell-gene-expression/software/overview/welcome (accessed on 8 April 2022)) on the original data to identify the data filtering, alignment, quantitative, and recycling cells, finally, the gene expression matrix of each cell was obtained. The number of cells obtained by the CellRanger-based cell calling algorithm is 14,113. The specific sample sequencing data statistical results are shown in Supplementary file S3.

#### 2.4.2. Advanced Analysis of Single-Cell Data Based on Seurat

The Seurat packet (https://satijalab.org/seurat/ (accessed on 1 October 2022)) in R was then used for cell filtration and analysis. We set the condition of cell filtration as: the number of genes identified in single cells greater than 500 and fewer than 30,000; the total number of UMI detected in a single cell greater than 1000; the proportion of mitochondrial gene expression in single cells less than 25%; use the DoubletFinder package to remove multicellular cells. After screening, 10,374 cells were obtained. The follow-up for further filtering, standardization, cell subgroup classification, each subgroup analysis, and marker gene screening of differentially expressed genes was performed by Seurat.

### 2.5. Drawing of Pie Chart and Histogram

The analysis of single and double pie charts was performed using the OmicShare tools, a free online platform for data analysis (https://www.omicshare.com/tools (accessed on 12 October 2022)). The histogram analysis was performed using the OmicStudio tools at https://www.omicstudio.cn/tool (accessed on 14 October 2022). The single-cell pie chats were drawn by the ggpie and ggplot2 R package.

### 2.6. Data Sets of Carotid Atherosclerosis

The gene expression profiles for carotid atherosclerosis were found in the GSE159677 dataset of the Gene Expression Omnibus database (GEO), which may be accessed at http://www.ncbi.nlm.nih.gov/geo/ (accessed on 10 May 2022). Single-cell specificity analysis and mapping were similar to those described above in femoral artery plaques. 

### 2.7. Data Acquisition and Preprocessing

The RNA-seq transcriptome data of patients with atherosclerosis and clinical traits were obtained from GEO. The GSE100927 dataset consists of 104 patients with atherosclerotic carotid artery tissues, atherosclerotic femoral artery tissue, atherosclerotic infra−popliteal artery tissue, and corresponding normal artery tissue and was compiled on the GPL20795 (Illumina HiSeq X Ten Homo sapiens) platform. Data were normalized using R statistical software. Principle component analysis (PCA) was used to explore whether there were differences between carotid and femoral atherosclerotic artery tissues. All data used in the study were obtained from the GEO; hence, ethics approval and informed consent were not required.

### 2.8. Gene Set Pathway Enrichment Analysis

We downloaded the 50 hallmark gene sets, necroptosis gene set, pyroptosis gene set, and ferroptosis gene set from the GSEA database, composing the list of gene sets. Then, the ssGSEA algorithm in the GSVA package of the R software (http://www.bioconductor.org/packages/release/bioc/html/GSVA.html (accessed on 10 September 2022)) was used to calculate the enrichment scores of every pathway in every sample. Next, we used the limma R package to obtain differential pathway enrichment scores between carotid-atherosclerotic tissue and femoral-atherosclerotic tissue [15]. Finally, we compared the scores of necroptosis pathways in different tissues and genders.

### 2.9. Immunofluorescence Staining

The atherosclerosis tissue underwent standard saline irrigation, 4% paraformaldehyde fixation, and graded ethanol dehydration. Endogenous peroxidase was inhibited by 3% H_2_O_2_ while the sections were submerged in ethylenedia-minetetraacetic acid (EDTA) antigen retrieval buffer. After blocking with 3% bovine serum albumin (BSA), the sections were incubated with CLEC10A antibody (Servicebio, GB114760, 1:500), F13A1 antibody (Servicebio, GB113293, 1:2400), LGALS3 antibody (Servicebio, GB111145, 1:1000), S100A8 antibody (Servicebio, GB11421, 1:1500), SPP1 antibody (Servicebio, GB112328, 1:500), and TPSAB1 (Servicebio, GB12110, 1:500) overnight at 4 °C. The secondary antibody was incubated for 50 min at room temperature.

## 3. Results

### 3.1. Single-Cell Analysis of Femoral Atherosclerosis

**Figure 1:**
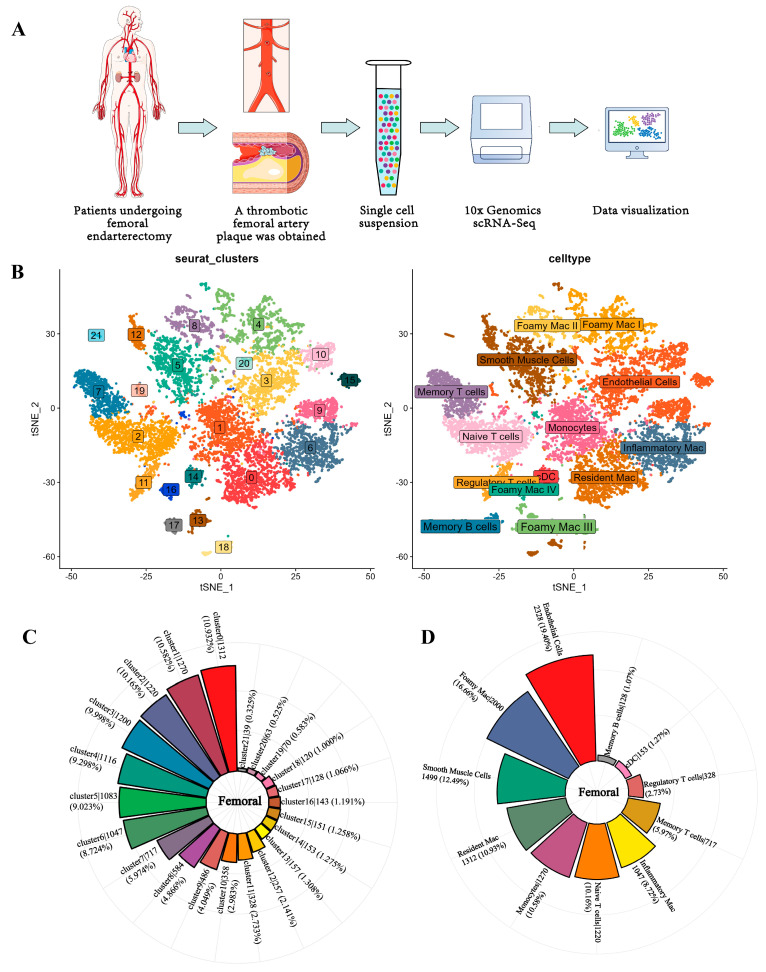
Procedures for sampling and analysis of femoral atherosclerotic plaques. (**A**). Femoral atherosclerotic plaques were obtained by femoral endarterectomy, and approximately 14,000 cells were extracted for subsequent analysis. (**B**). The tSNE projection cluster scatter diagram was noted with clusters and cell types. (**C**). The number and percentage of each clustered cell type based on T-distributed stochastic neighbor embedding (tSNE) projection are presented. (**D**). The pie chart shows the proportion of all cell types. Less to more in a clockwise direction.

The samples of femoral-artery plaques were obtained by femoral endarterectomy and were quickly placed in the tissue storage solution and immediately sent for single-cell sequencing (Figure 1A). Based on the genome map and detailed statistics for unique molecular identifiers, the expression levels of the genes in each cell were calculated (Supplementary file S4). Reportedly, 14,113 cells from the patient were taken. All cells’ gene expression variability was subjected to principal component analysis, and the cells were subsequently divided into cell-type groups using graph-based clustering of the informative principal components (*n* = 8). Twenty-one groups were found using graph-based clustering, as shown in Figure 1B. The ratio of cells in each cluster decreases from cluster 1 to 14 (Figure 1C).

The top 50 expressed genes (fold change) in each cluster were matched with known indicators. Thus, we discovered clusters of cells that could be simply ascribed to established cell lineages using standard marker genes. We identified 14 cell types, including resident macrophages (F13A1), monocytes (CD52, FCN1, VCAN, S100A9, S100A8), naive T cells (IL7R, CCR7), endothelial cells (SPARCL1, VWF, SPP1, EDN1), Foamy Mac I (CD9, TREM2, APOC1, GPNMB, FABP5), smooth muscle cells (COL1A2, COL1A1, TAGLN), inflammatory mac (CXCL2), memory T cells (IL32, SELPLG, TNFSF10), Foamy Mac II (FABP5, GPNMB, TREM2, APOC1, CD9, SPP1), regulatory T cells (FOXP3), Foamy Mac III (CD9, SPP1), conventional dendritic cell (cDC) (CD1C, CLEC9A, FCER1A, CLEC10A, IRF8), Foamy Mac IV (CD9, TREM2, GPNMB, SPP1, APOC1), and memory B cells (NCR3, CD27, CD86) (Figure 1B). The number of each cell type is shown in counterclockwise order from most to least in Figure 1D. The number of endothelial cells was the highest, the number of foam cells in immune cells was the highest, and the number of memory B cells was the lowest.

### 3.2. Single-Cell Analysis of Carotid Atherosclerosis

We downloaded single-cell data for six human carotid plaque samples from the GEO database, including three samples close to the core plaque and three from the lipid core plaque. To match the pathological composition of the femoral artery plaque, we also analyzed six carotid artery samples instead of the core or the samples away from the core. Using the Seurat package in R, we divided single cells into groups and performed mapping on them.

**Figure 2:**
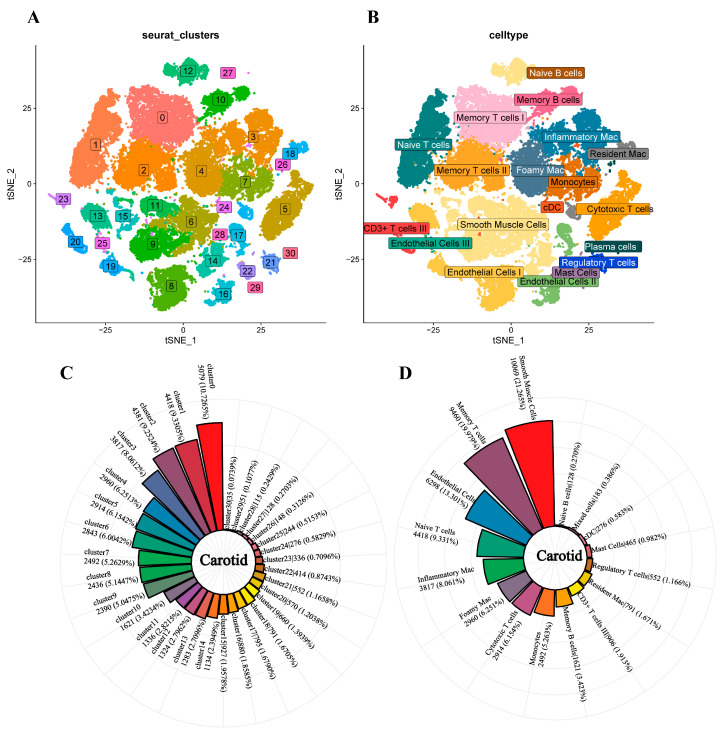
The single-cell analysis of carotid plaque. (**A**). Thirty-one clusters were noted with different colors in the dimplot based on tSNE projection (**B**). The tSNE projection cluster scatter diagram was noted with cell type. Eighteen cell types were identified and labeled by matching marker genes that were the same as those in the femoral plaques. (**C**). The number and percentage of each cluster based on tSNE projection is presented. The number in cluster0 was the most, and cluster 30 was the least. (**D**). The pie chart shows the proportion of all cell types. More to less in a clockwise direction.

Before single-cell data mining, we filtered dead or stressed cells by Seurat based on some indicators as follows: (1) the number of genes detected in a single cell is greater than 500 and fewer than 30,000. (2) The total number of UMI detected in a single cell is greater than 1000. (3) The percentage of mitochondrial gene expression in a single cell is less than 5. After unqualified cells and genes were filtered, there were still 47,350 cells and 26,755 genes. Then, 47,350 cells were grouped into 31 cell populations by the tSNE (Figure 2A,C). We identified the top five genes for each cluster with the highest pvalue (Supplementary file S5). In order to maintain the consistency of cell types identified, the marker genes used by femoral single-cell analysis mentioned previously were also compared with the top 50 carotid artery marker genes, and finally, 19 cell types were identified, including memory T cells I (IL32, TNFSF10, SELPLG), naive T cells (IL7R), memory T cells II (IL32), Foamy Mac (SPP1, FABP5, APOC1), cytotoxic T cells (GZMK, PRF1), smooth muscle cells (COL1A2, COL1A1, TAGLN), monocytes(S100A8, S100A9, FCN1), endothelial cells I (PLVAP), memory B cells(NCR3, CD27, CD86), endothelial cells II (EDN1, VWF, SULF1), resident macrophages (F13A1, LYVE1), CD3+ T cells (FGFBP2, PRF1, GZMB), regulatory T cells (LGALS3), mast Cells (TPSAB1 HDC), cDC2 (CLEC10A), inflammatory mac (CXCL2), naive B cells (IGHM, IGHD), and plasma cells (IGHG1) (Figure 2B,D).

### 3.3. Analysis of Immune Cells of Carotid and Femoral Atherosclerosis

**Figure 3:**
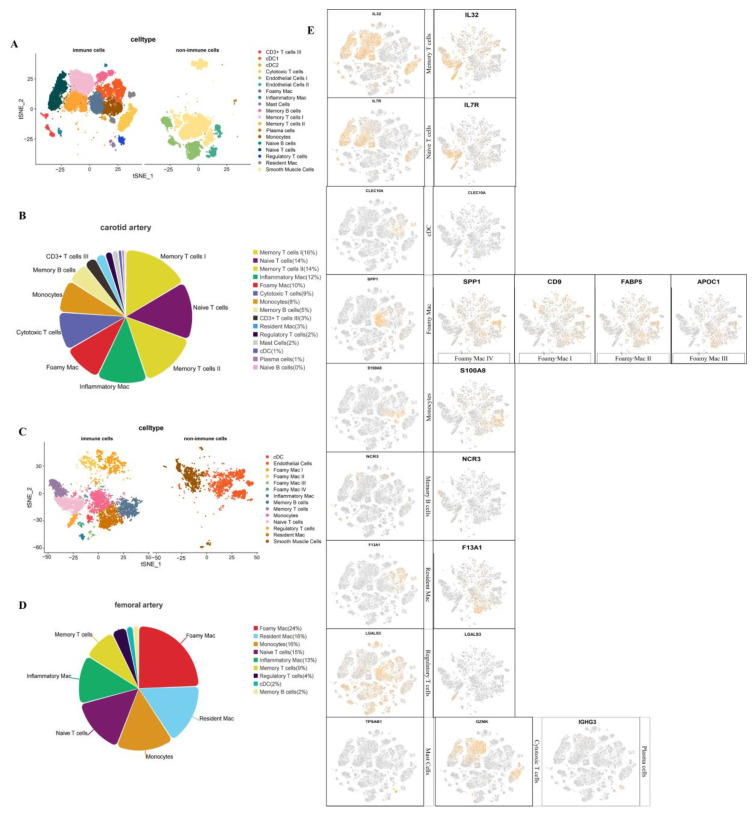
(**A**,**C**) are cluster plots of immune cells isolated from carotid and femoral atherosclerotic plaques, respectively. (**B**,**D**) are the proportions of each immune cell in the carotid and femoral arteries, respectively. In the two plots, yellow is for memory T cells, purple for natural T cells, green for inflammatory macrophages, red for foam cells, cyan gray for killer T cells, orange for monocytes, light yellow for memory B cells, black for CD3 T cells, sky blue for resident macrophages, dark purple for regulatory T cells, gray for mast cells, cyan represents cDC cells, burgundy represents plasma cells, and pink represents natural B cells. The genes used by the two plaques to identify cell types are the same. (**E**) shows the marker gene of each cell type and the distribution of this marker gene in the cluster map. Among them, cytotoxic T cells, mast cells, and plasma cells were only found in carotid plaques, which we marked at the end of the picture. There are four types of foam cells in the femoral artery.

In order to further observe the immune cell composition of the femoral artery and carotid artery plaque more intuitively, first, we separated the immune cells and divided all identified cells into immune cells and non-immune cells (Figure 3A,C). In the adaptive immunity response, T cells recognized four types of T cells, including regulatory T cells, naive T cells, memory T cells, and cytotoxic T cells (CD3 + T cells were only present in endothelial-like samples), and B cells recognized two types, including natural B cells and memory B cells. The identified innate immunity cells included cDCs, mast cells, monocytes, and macrophages at different stages (foamy macrophages, resident macrophages, inflammatory macrophages) (Figure 3B,D). The cell types of femoral plaques were basically the same as those of carotid plaques. The specific immune cells of two types of plaques both include B cells and T cells. However, the subtypes in T cells of the femoral do not include cytotoxic T cells compared with the carotid plaque. The innate immunity cells did not include mast cells, which were identified in the carotid plaque (Figure 3B). Markers for each cell subtype, as well as cell types for both plaques, are shown in Figure 3E. In this figure, the marker genes used to discriminate cell types are the same in both plaques. The cell types identified by the two plaques are basically similar.

### 3.4. Differences in the Proportion of Immune Cell Infiltration between Femoral and Carotid Plaques

**Figure 4:**
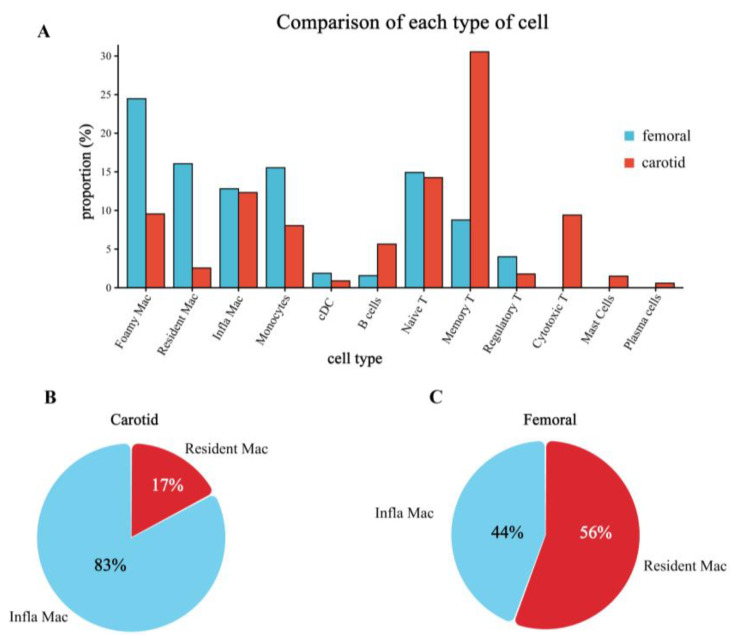
Comparison of immune cells between carotid and femoral plaques. (**A**). Cell percentage changes of each cluster in carotid and femoral samples are shown. The red bar represents carotid arteries. The blue bar represents femoral arteries. (**B**) The pie chart shows the proportions of resident macrophages and inflammatory macrophages in the carotid. (**C**) The pie chart shows the proportions of resident macrophages and inflammatory macrophages in femoral samples. Blue represents inflammatory macrophages. Red represents resident macrophages.

We calculated the proportion of each immune cell in total immune cells. T cells were the most infiltrated immune cells in the carotid artery, while macrophages were the most infiltrated immune cells in the femoral artery. In order to more intuitively show the difference between the two plaques, we compared the proportion of each immune cell in the two plaques, as shown in Figure 4A. Except for regulatory T cells and naive T cells, other types of adaptive immunity were more prevalent in carotid arteries and less prevalent in femoral arteries. Innate immunity cells were more in the femoral artery but fewer in the carotid artery.

The infiltration ratio of immune cells may change the plaque stability and eventually lead to the rupture of the arterial plaque. The most widely studied and well-known is the proportion of macrophage subtypes. Therefore, we then compared the proportion of inflammatory macrophages with the number of resident macrophages (Figure 4B) and found that the proportion of inflammatory macrophages was significantly higher in the carotid artery than in the femoral artery.

### 3.5. Successful Validation of the Difference in Cell Proportion between Carotid and Femoral Arteries

**Figure 5:**
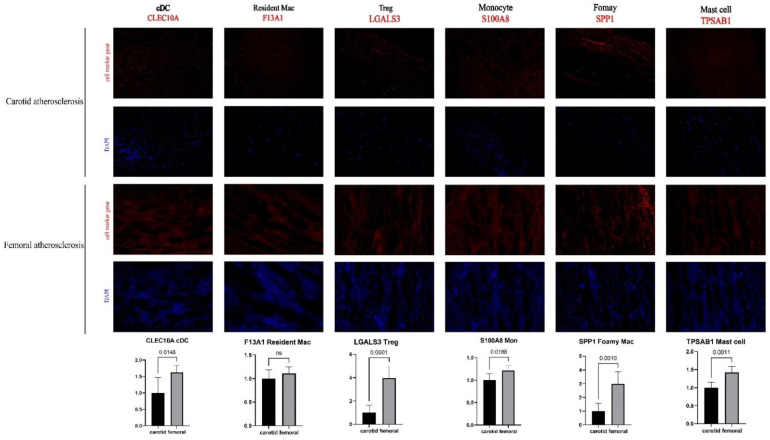
Validation of the difference in immune-cell count through immunofluorescence staining. cDCs were labeled by CLEC10A. Resident macrophages were labeled by F13A1. Regulatory T cells were labeled by LGALS3. Monocytes were labeled by S100A8. Foamy macrophages were labeled by SPP1. Mast cells were labeled by TPSAB1.

To further verify the differences in the proportions of various immune cells in carotid and femoral atherosclerotic plaques, we used their marker genes in the single-cell analysis (Figure 3E) to observe cDC, resident macrophages, regulatory T cells, monocytes, foamy cells, and mast cells in the two atherosclerotic tissues by fluorescent immunostaining (Figure 5). By observing the immunofluorescence staining and semi-quantitative analysis, we could see significant differences between the carotid and femoral arteries (cDC, *p* = 0.0148; regulatory T cells, *p* < 0.0001; monocytes, *p* = 0.0188; foamy cells, *p* = 0.001; mast cells, *p* = 0.0011). Immunofluorescence staining for resident macrophages shows no statistically difference between carotid and femoral samples, but the trend to increased numbers of cells in femoral arteries. The results were consistent with the above analysis in single cells (Figure 4A).

### 3.6. Bulk Analysis of Two Types of Atherosclerosis

**Figure 6:**
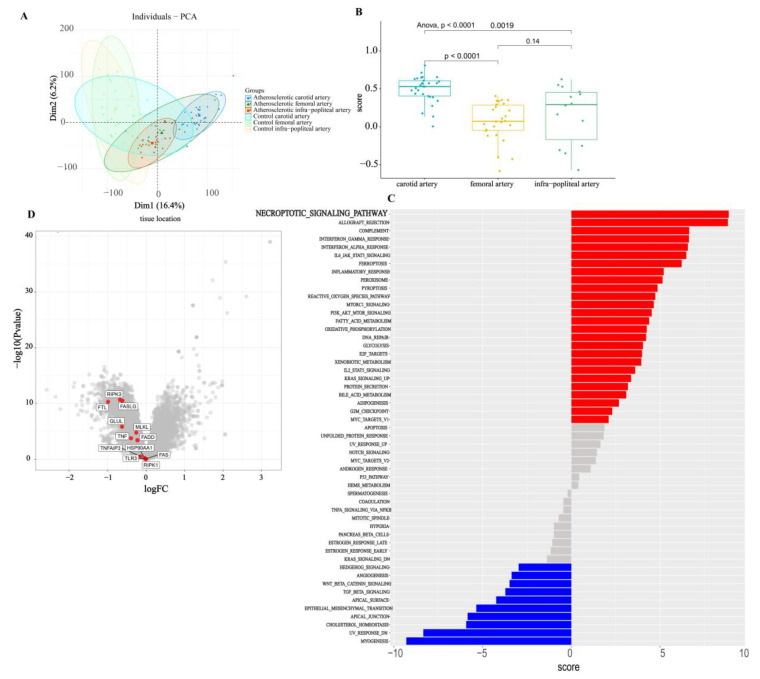
Results of transcriptome analysis of carotid and femoral artery samples. (**A**). PCA clustering results for carotid, femoral, and popliteal arteries. (**B**). Statistically significant differences were found in the immunoscores of carotid, femoral, and popliteal arteries. (**C**). The results of immunoscores for carotid and femoral samples are shown. The red items exhibit the more incredible immune response in the carotid samples. The blue color was more common in femoral samples. (**D**) The differentially expressed genes between femoral and carotid samples are represented in a volcanic map. Necroptosis-related genes are labeled in the figure.

Although the single-cell research level is more accurate, it cannot view the occurrence and development of diseases at a macro level. The mRNA transcriptome is not accurate to the cellular level, but it can screen out the prominent genes and mechanisms to find the key mechanism of the occurrence of diseases. To better observe the primary and secondary relationship of immune cells in carotid and femoral plaques, we downloaded the atherosclerosis dataset from GEO, including 27 femoral arteries and 28 carotid arteries. The carotid and femoral transcriptome gene expression profiles were compared. First, through PCA clustering, it was found that the samples of the femoral artery and carotid artery were, respectively, clustered in the two circles of the cluster map (Figure 6A). Although all of them were atherosclerotic plaques, there were still some differences between the samples. The immunoscores of carotid and femoral plaques were significantly different (Figure 6B). We compared the gene set enrichment analysis (ssGSEA) scores of the carotid artery and the femoral artery, and found that the necroptosis pathway had the highest t-value. (Figure 6C). We identified gene expression differences between femoral and carotid groups and found that genes related to necroptosis were highly expressed in the carotid artery and minimally expressed in the femoral artery. The expression of FLT, RIPK3, and GLUL was significantly different (*p* < 0.05, |log2FC| > 0.5) (Figure 6D).

## 4. Discussion

With aging, calcification of the vascular wall, deterioration of elasticity, endothelial cell damage, and cardiac function decline are inevitable and irreversible phenomena, leading to severe cardiovascular death events [16]. The most common one is atherosclerosis, in which excessive lipids from the peripheral circulation are deposited in the subendothelial layer of the artery, causing a series of immune and inflammatory cells such as macrophages and neutrophils to gather around the lesion and form atherosclerotic plaques, resulting in local stenosis of the blood vessels [17]. Thinning of the fibrous cap on the surface will lead to the exposure of the formed lipid plaque from the subendothelium, forming an embolism and causing a distal occlusion. The better-known atherosclerosis is coronary and carotid atherosclerosis. Coronary atherosclerosis can lead to myocardial ischemia, resulting in stable angina pectoris and even myocardial infarction at the late stage. Unstable carotid atherosclerosis can lead to cerebral infarction. Much of the study of atherosclerosis has revolved around mechanisms in the carotid and coronary arteries. Severe femoral artery stenosis can also lead to gangrene, ulcer, and even necrotic amputation of the lower limbs, which seriously affects the quality of life [18]. However, the early symptoms of femoral artery stenosis are not obvious, and it is prone to misdiagnosis and delay. Therefore, it is of great significance to study the mechanism of atherosclerotic plaque in the femoral artery and its heterogeneity with atherosclerosis in other parts of the body.

This paper is the first article on femoral atherosclerosis single-celled sequencing. The previous studies mainly used immunofluorescence microscope to observe the cell types and counts in femoral atherosclerosis tissue [19,20]; hence so far, no one article’s complete description explored the artery atheromatous plaque cell composition and proportion. According to single-cell sequencing, the femoral atherosclerotic plaque is mainly composed of resident macrophages, inflammatory macrophages, foam cells, monocytes, natural T cells, regulatory T cells, memory T cells, B cells, and two types of non-immune cells, smooth muscle cells and endothelial cells. Among them, endothelial cells accounted for the largest proportion, immune cell foam cells accounted for the largest proportion, followed by smooth muscle cells and resident macrophages. Immune cells accounted for more than non-immune cells, and the corresponding non-immune cells were less numerous.

Although both carotid and femoral arteries are arteries, there are obvious differences in atherosclerosis’s pathogenesis and pathological manifestations. Shaikh et al. observed the results of T cells and macrophages under immunohistochemistry in the carotid artery and femoral artery atherosclerotic plaques and found that the number of T cells and macrophages, as well as the number of M1 and M2, were significantly different. There were also marked differences in the expression of inflammatory factors between carotid and femoral plaques. The expression of matrix metalloproteinase-2 and matrix metalloproteinase-9 in the carotid artery was significantly higher than that in the femoral artery. Meanwhile, there was more fibrous connective tissue in the femoral artery than in the carotid artery. Herisson et al. collected 88 plaques and found that most of the pathological tissue of carotid atherosclerotic plaques was fibrous cap calcification, which was more likely to exhibit plaque rupture [21]. At the same time, the femoral artery was mainly fibrous calcified plaque, which was more stable. According to the results of carotid atherosclerosis analyzed in this paper, smooth muscle cells accounted for the largest proportion of non-immune cells. In contrast, memory T cells accounted for the largest proportion of immune cells, followed by endothelial cells and natural T cells. By comparing the proportion of cells in the carotid artery and the femoral artery, it was found that the proportion of T cells in the carotid artery was much higher than that in the femoral artery. Previous sequencing of carotid atherosclerotic plaques also proved that the number of T cells in the carotid artery was the largest, and T cells could also affect the stability of arterial plaques(KLRC1, CXCR3, STAT3, IFNGR1, HLA-B) and chemotaxis functions (CCL5, CCL4, CXCR6) [22,23]. The liver metabolizes lipids and T cells, including regulatory T cells, Th1, Th2, and Th17 under the stimulation of high blood lipids. Then, they are released into the peripheral circulation and finally enter the atherosclerotic plaque to activate local adaptive immunity [24,25,26]. Although T cells do not accumulate large amounts of oxLDL subcutaneously similar to macrophages, they are cytotoxic and secrete pro-inflammatory factors locally. For example, CD8 + T cells locally overexpress transcripts associated with T cell activation (NFATC2, FYN, ZAP70), cytotoxicity (GZMA, GZMK), and T cell failure (EOMES, PDCD1, LAG3). CD4 + T cells overexpressed transcripts with proinflammatory functions (KLRD1, KLRC1, CXCR3, STAT3, IFNGR1, HLA-B) and chemotaxis functions (CCL5, CCL4, CXCR6) [27,28,29]. The number of regulatory T cells decreased in atherosclerosis, and the expression of several genes related to immunosuppression decreased significantly [24]. In this paper, the carotid artery exhibited more CD4+T cells with pro-inflammatory properties and CD8 + T cells with cytotoxic properties, while regulatory T cells expressing immunosuppression-related genes were, on the contrary, fewer in the carotid artery and more in the femoral artery, which also indicated that carotid atherosclerotic plaques were more unstable and tended to rupture compared with the femoral artery. 

Under the stimulation of oxLDL and apolipoprotein, monocytes enter the endodermis from peripheral circulation and differentiate into different macrophage phenotypes: some exhibited greater oxLDL uptake and an increased propensity to form foam cells; some overexpressed inflammatory factors such as TNF-α and IL-6 and matrix metalloproteinases MMP9 and MMP2, which increased the local inflammatory response and made the fibrous cap thin [30]. In addition, some macrophages come from macrophages residing in vascular tissues, and their expression of pro-inflammatory factors IL-1 and IFN is significantly reduced, mainly to inhibit the occurrence of the inflammatory response. In this study, we found that there were more resident macrophages than inflammatory macrophages in femoral atherosclerosis, but there were nearly five times as many inflammatory macrophages as resident macrophages in the carotid artery. The proportion of inflammatory macrophages and resident cells in local infiltration has a critical effect on the stability of atherosclerotic plaques and the outcome of cardiovascular events [31]. This result again confirms the vulnerability of carotid plaque compared to femoral plaque.

Therefore, by analyzing the cellular infiltrating components and proportions of the two types of atherosclerosis, the prognosis of atherosclerosis is not limited to conventional risk factors (such as dyslipidemia, hypertension, and diabetes) but is also related to the infiltration and release of cellular and inflammatory factors.

Our study compared the differences in immune cell infiltration between carotid and femoral arteries from the perspective of single-cell sequencing, which could provide a better target and basis for the treatment of atherosclerosis by targeting macrophages or T cells. However, the selected patient was on anticoagulant drugs, so its influence on gene expression could not be ruled out. Thus, more studies, including in vivo studies and randomized controlled trials, are required to clarify these effects.

## Data Availability

Not applicable.

## Supplementary Materials

The following supporting information can be downloaded at: https://www.mdpi.com/article/10.3390/jcdd9120465/s1, Supplementary file S1: Detailed clinical information for patient samples.; Supplementary file S2: Detailed documentation of single-cell quality control; Supplementary file S3: Sequencing data statistical results; Supplementary file S4: The gene-expression levels of each scRNA-seq cell; Supplementary file S5: The heatmap of top five genes for each cluster.
