# Supplementary figures and images for: A Single-Cell Atlas of the Atherosclerotic Plaque in the Femoral Artery and the Heterogeneity in Macrophage Subtypes between Carotid and Femoral Atherosclerosis
Source: J Cardiovasc Dev Dis. 2022 Dec 16;9(12):465. doi: 10.3390/jcdd9120465 (PMC9788114; doi:10.3390/jcdd9120465)

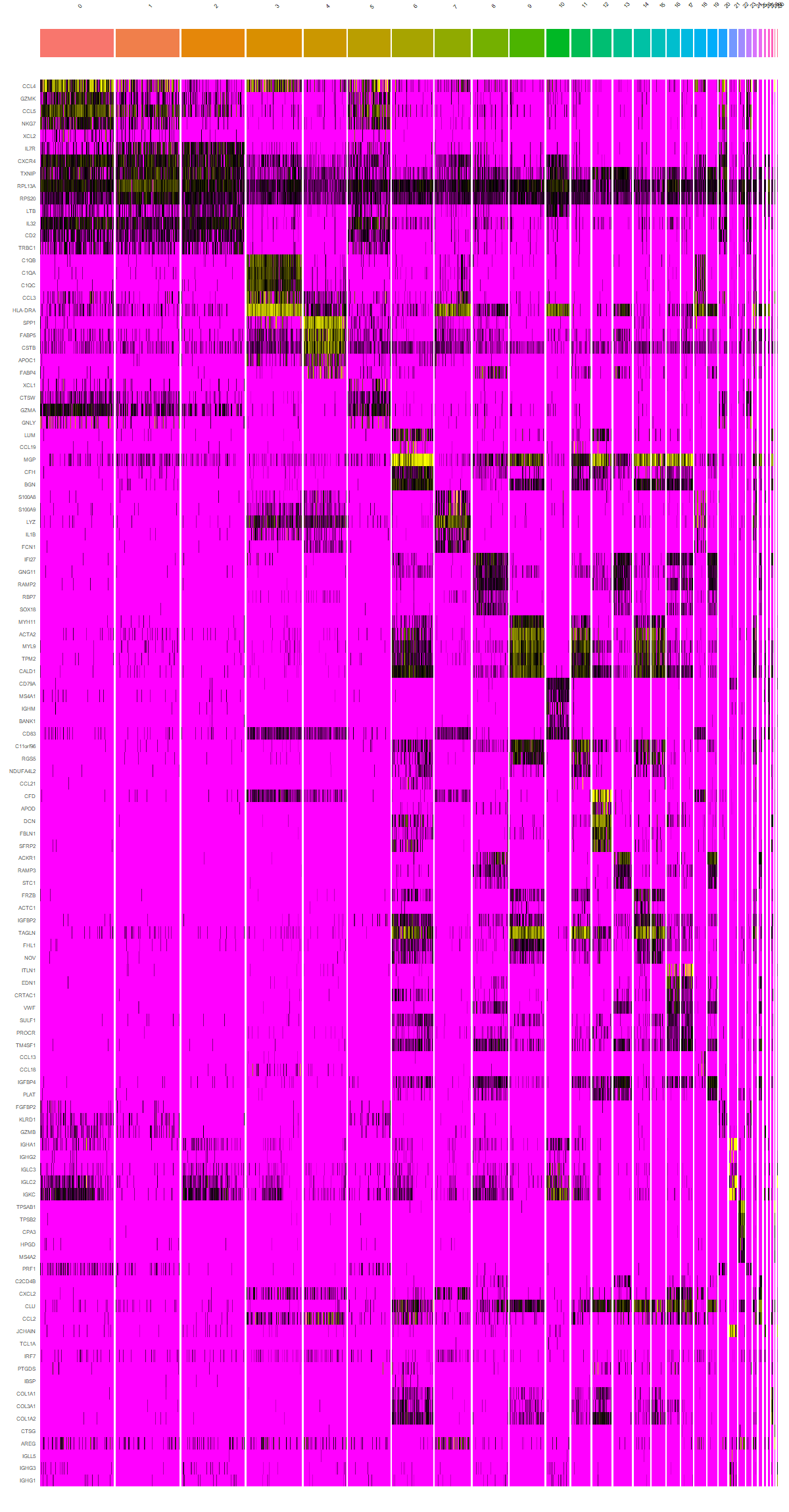

Supplement: Supplementary file 1 [file jcdd-09-00465-s001.zip › supplementary file S5.tiff]
